# Clinical Response to a PARP Inhibitor and Chemotherapy in a Child with BARD1-Mutated Refractory Neuroblastoma: A Case Report

**DOI:** 10.21203/rs.3.rs-3250117/v1

**Published:** 2023-08-16

**Authors:** Maggie Cupit-Link, Kohei Hagiwara, Jinghui Zhang, Sara M. Federico

**Affiliations:** 1 Department of Oncology, St. Jude Children’s Research Hospital, Memphis, TN 38105; 2 Department of Computational Biology, St. Jude Children’s Research Hospital, Memphis, TN 38105

**Keywords:** case report, PARP inhibitor, BARD1, neuroblastoma, high-risk

## Abstract

Despite advances in the treatment of high-risk neuroblastoma, approximately half of these patients die from the disease. Targeted therapy based on synthetic lethality associated with homologous recombination deficiency (HRD) caused by germline mutations in homologous recombination repair genes has shown great efficacy in several adult solid tumors. Here we report the first successful treatment of a pediatric patient with refractory neuroblastoma and a germline pathogenic mutation in *BARD1* using a PARP inhibitor, talazoparib, in combination with cytotoxic chemotherapy and radiation therapy. Allele-specific expression in RNA-seq indicates bi-allelic loss of *BARD1* in tumor; however, the HRD score was below the threshold currently used for HRD classification in adult cancers. Our study demonstrates that the use of PARP inhibition in combination with DNA-damaging agents should be considered in children with *BARD1*-mutated neuroblastoma and cautions against the use of HRD score alone as a biomarker for this pediatric population.

Neuroblastoma is the most common extracranial solid tumor of childhood with broad phenotypic variability^1^. Patients with relapsed or refractory high-risk neuroblastoma (HR-NBL) have a poor prognosis^2^. Recently, the combination of chemotherapy with an anti-GD2 monoclonal antibody (mAb) demonstrated a positive objective response rate with 41.5% (relapsed) and 32.3% (refractory) of patients responding to therapy^3^. However, little is known about the long-term outcome and durability of response following this regimen. Patients often experience progressive disease while on treatment or subsequent relapses; thus, alternative therapeutic options are needed.

Studies of genome-wide association (GWAS) and mutational screening of cancer predisposition genes in large cohorts have identified GWAS polymorphic variants and pathogenic/likely pathogenic (P/LP) germline mutations, respectively, in *BARD1*^4–17^. *BARD1* encodes the BRCA1-associated RING domain 1 protein which forms the BRCA1-BARD1 heterodimer to repair DNA double-strand breaks by homologous recombination. Loss of *BARD1* has been shown to confer homologous recombination repair (HRR) deficiency in adult cancer cell lines^18^. This defect has been effectively exploited in the treatment of adult *BRCA*-mutated breast and ovarian cancers through treatment with poly-ADP-ribose-polymerase inhibitors (PARPi) ^19,20^. More recently, the FDA approved olaparib, a PARPi, for treating metastatic castration-resistant prostate cancer harboring mutations in HRR genes including *BARD1*^21^. Thus, we postulate that neuroblastoma patients with *BARD1* mutations may also benefit clinically from PARP inhibition.

To date, two phase 1 clinical trials (ADVL1411, NCT02116777 and BMNIRN, NCT02392793) conducted in the pediatric patient population have evaluated the PARPi talazoparib in combination with temozolomide and/or irinotecan^22–24^. The combinations were safely administered and both trials determined the recommended phase 2 doses of the combinations. Frequent toxicities included hematologic and gastrointestinal toxicities^22–24^. A subset of patients with Ewing sarcoma treated with the combination of talazoparib plus irinotecan with or without temozolomide demonstrated a clinical response^23^. However, only one patient with neuroblastoma was treated on these trials.

Here we report the first sustained response of a pediatric patient with refractory neuroblastoma and a *BARD1* germline pathogenic mutation to a therapeutic regimen including a PARPi (talazoparib) in combination with irinotecan and radiation therapy. We further examined the allelic state of the mutation as HRD and response to PARPi are often associated with bi-allelic not mono-allelic loss of HRR genes^25–27^. Additionally, we compared the HRD score of our index patient with 58 other pediatric patients with neuroblastoma profiled by our Clinical Genomics program (**Supplementary Table S1**).

The patient was a 22-month-old female with a history of a congenital ventricular septal defect. She presented to a local hospital with a one-month history of intermittent fevers, leg pain, and refusal to ambulate. She was empirically treated for infectious endocarditis and subsequently evaluated for a malignancy. A workup identified a left adrenal mass and widespread lymph node, bone, and bone marrow metastases. The patient received 5 cycles of Induction chemotherapy as per SIOPEN N7^28^, followed by surgical resection of the left adrenal primary tumor and retroperitoneal nodal disease. She continued to have persistent bone marrow disease and received 2 cycles of post-operative cyclophosphamide and topotecan and 3 cycles of irinotecan and temozolomide. For Consolidation therapy, the patient received a single autologous stem cell transplant with a busulfan/melphalan conditioning regimen and 21 Gy of radiation therapy to the primary tumor bed. Post-Consolidation therapy consisted of 6 cycles of cis-retinoic acid. At the completion of therapy, 21 months after diagnosis, the patient developed progressive bone marrow disease with 30% involvement of the bone marrow and was referred to our institution for additional therapy.

The patient was subsequently treated in our hospital as outlined in Fig. 1A. On arrival to our facility, the patient received 2 cycles of irinotecan, temozolomide, and the anti-GD2 monoclonal antibody (mAb) dinutuximab, as per the Children’s Oncology Group study ANBL1221^3^. The patient’s bone marrow involvement improved to 10%, which qualified as stable disease per the revised International Neuroblastoma Response Criteria^29^. Treatment was changed, and the patient received 2 cycles of cyclophosphamide, topotecan, and dinutuximab. The bone marrow continued to demonstrate 10% involvement. Concurrently, results from whole exome (WES) and transcriptome sequencing (RNA-seq), which had been performed on the germline and formalin-fixed paraffin embedded (FFPE) tumor obtained at the time of diagnosis as part of our clinical genomic sequencing (ClinGen) program^30^, identified a pathogenic germline mutation in exon 4 of *BARD1* causing protein truncation by frameshift (S179_Y180fs). This frameshift mutation matches a known pathogenic ClinVar variant (VCV000186576) ^31^ previously found in families with breast and ovarian cancer^32,33^.

The therapeutic regimen was changed to the combination of talazoparib and irinotecan, as per BMNIRN^23^, although the patient was not enrolled on a clinical trial. This decision was due to the following: 1) the patient’s persistent disease in the bone marrow (10%), 2) lack of available stem cells to support therapeutic meta-iodobenzylguanidine (MIBG) therapy, 3) inability to meet eligibility criteria for alternative clinical trials due to thrombocytopenia, and 4) data supporting use of PARPi therapy in patients with HRD-mutated cancers. The patient received 2 cycles of therapy (talazoparib 400mcg/m^2^ IV, days 1–7 and irinotecan 40mg/m^2^ IV days 1–5, 21-day cycles) with myeloid growth factor and platelet infusions. Of note, the administered talazoparib dose was different from the recommended phase 2 dose (talazoparib 600mcg/m^2^ IV, days 1–6) due to the lack of a liquid formulation and limited capsule dosages, and adherence was reported by the patient’s guardians. The patient’s bone marrow was negative for tumor cells following 2 cycles of therapy and has remained negative since then. Following cycle 5, the irinotecan dose was decreased to 20mg/m^2^ IV days 1–5 and was then eliminated after cycle 6. The patient continued to receive talazoparib monotherapy (due to grade 4 thrombocytopenia attributed to irinotecan). The treatment was well-tolerated. A FDG-PET scan obtained after cycle 18 continued to demonstrate mild avidity in the calvarium, left rib and left femur. The bone marrow exam was negative. Single agent talazoparib was continued, and the patient received concurrent radiation therapy to the calvarium (30.6 Gy), left rib (21 Gy), and left femur (21 Gy). Talazoparib was discontinued after 26 cycles. The patient’s last bone marrow evaluation was performed at the 3 months off therapy evaluation and was negative for disease. The patient has now been asymptomatic and off therapy for 22 months without evidence of disease.

The response to talazoparib prompted us to investigate homologous recombination deficiency (HRD) in the patient’s tumor, which is associated with bi-allelic pathogenic alterations on HRR genes^34^. However, the variant allele fraction (VAF) of the pathogenic mutation S179_Y180fs in tumor was 0.36 (read count: 5/14), indicating heterozygosity. This was consistent with the absence of loss of heterozygosity (LOH) and copy number variation (CNV) from tumor WES. Interestingly, the VAF of this mutation in RNA-seq was 0.8, raising the possibility of epigenetic silencing as the second hit. Limited by lack of a biospecimen for epigenetic profiling, we resorted to evaluating allele-specific expression (ASE) of *BARD1*, an approach we employed previously to determine a second hit on *TP53* in a tumor^35^. By incorporating VAF of the four heterozygous germline SNPs in *BARD1* from WES along with the S179_Y180fs mutation, ASE was detected (*p* = 0.015, Methods), projecting a complete loss of the wildtype *BARD*1 transcript after adjusting for the 60% estimated tumor purity (Fig. 1B).

Importantly, the low *BARD1* expression of our index tumor (< 2 fragments per kilobase of transcript per million mapped reads) amongst the 59 ClinGen neuroblastoma samples is consistent with reduced expression expected from epigenetic silencing possibly coupled with a nonsense-mediated decay (Fig. 1C, left). Intriguingly, the HRD score of this tumor, estimated from scarHRD^36^, is only 21 lower than the widely adopted threshold of 42 for determining HRD status^37^. When examining HRD score in all 59 neuroblastoma samples, which include 3 cases with P/LP germline mutations in HRR genes (Methods), we found that only one neuroblastoma harboring a pathogenic germline *CHEK2* splice site mutation exceeded this threshold (Fig. 1C, right).

Our study presents the successful treatment of a pediatric patient with refractory *BARD1*-mutated HR-NBL who achieved a sustained clinical response following treatment with talazoparib, irinotecan, and radiation therapy. To our knowledge, this is the first case report of a PARPi combination for the treatment of *BARD1*-mutated neuroblastoma. Although *BARD1* pathogenic mutations, which occur in approximately 1% of neuroblastoma patients^4^, are rare events, they may confer aggressive disease^5,38–40^. We demonstrate for the first time that bi-allelic loss of *BARD1* confers a targetable vulnerability in this disease. In our cohort of 59 NBLs, we found 3 cases with P/LP germline mutations in HRR genes (Figure 1 D), consistent with recent genomic profiling of HR-NBL germline and tumor samples, which showed multiple defective DNA repair mechanisms (*ATRX*, 11q LOH, *ATM*, *H2AFX*, *CHK1*, *CEP131* and *MRE11*) ^41–45^. Published studies of drug exposure experiments using *in vitro* cell line models and *in vivo* zebrafish models also suggest that patients with such defects may benefit from a similar therapeutic strategy^46–56^. Our study presents the first real-life clinical case which demonstrates that HR-NBL with HRD may be eligible for targeted agents, thereby reducing exposure to highly toxic nonspecific anti-cancer therapies which can potentially reduce late effects in the HR-NBL population.

Although HRD score has been broadly used as a predictor for response to PARP inhibition in the treatment of adult breast cancer, the low HRD score of our index NBL tumor suggests its limitation in predicting clinical response in pediatric NBL. Indeed, we observed a broad spectrum of HRD scores amongst the 3 NBLs with P/LP germline mutations in HRR genes (Fig. 1D). Thus, we caution against making clinical decisions solely based on HRD score, as our index patient would not have been eligible. This underscores the necessity of re-evaluating potential biomarkers for predicting therapeutic response in different cancer types and in the pediatric population. Specifically, HRD score, which was modeled on *BRCA-*mutated adult breast cancer, may not be an appropriate biomarker for predicting response to PARPi in the treatment of NBL or other pediatric cancers.

There are several limitations of the present study. First, this is a single patient case report, and validation in additional cases will be highly informative to future clinical trial design. Second, due to the sample limitation and overall low mutation rate of this case (10 somatic mutations by WES), we were unable to evaluate additional HRD-related mutational signatures which require somatic SNVs, indels and SVs detected by WGS to ensure statistical rigor in pediatric cancer.

In summary, our finding on the sustained complete response in a patient with refractory NBL supports future therapeutic studies assessing PARP inhibition with DNA damaging agents. Additionally, the lower-than-expected HRD score in our index patient will inspire either a recalibration of current HRD score or development of new biomarkers for predicting HRD in pediatric cancer which may require more broad use of WGS for exploring diverse types of mutational signatures.

## ONLINE METHODS

### Data Sources

This study was granted exemption by our Institutional Review Board in April 2023, classifying the project as Secondary Research (research involving only information collection and analysis involving the investigator’s use of identifiable health information when that use is for the purposes of research). Data about the patient’s case and clinical care were abstracted from the patient’s electronic medical record (EMR) at St. Jude Children’s Research Hospital (SJCRH). Photographs of bone marrow histology were taken by a pathologist.

The germline *BARD1* mutation was reported as part of the St Jude Clinical Genomics (ClinGen) program which employs three-platform whole-genome (WGS), whole-exome (WES) and transcriptome (RNA-seq) sequencing to report somatic and germline pathogenic/likely pathogenic variants. A modified version of a two-platform (WES and RNA-seq) pipeline was later developed for analyzing FFPE samples, which include our index patient sample (ID SJNBL031647). ClinGen genomic data from consented patients are uploaded to St. Jude Cloud^57^, where we obtained bam files (WES and RNA-seq) for all the samples used in this study.

### Analysis of Bi-allelic Loss of *BARD1*

**FIGURE 1:**
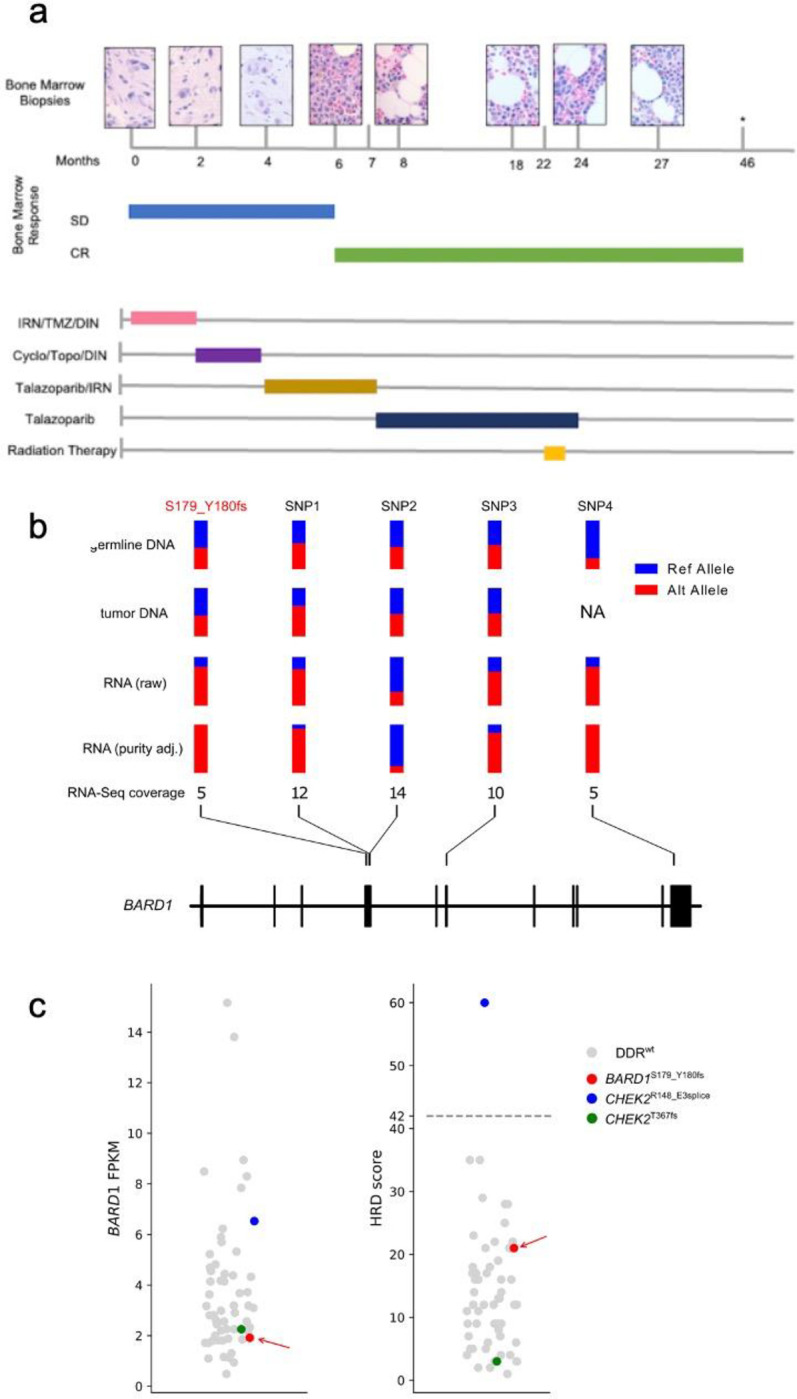
Treatment history and molecular features of a high-risk neuroblastoma patient with sustained response to PARP inhibitor. **a)** Index patient’s (patient ID SJNBL031647) bone marrow response over time in months, depicted by bone marrow biopsy histology and response per Revised INRC Criteria^29^, in correlation with treatments received over time. SD: stable disease, CR: complete response, IRN: irinotecan, TMZ: temozolomide, DIN: dinutuximab, Cyclo: cyclophosphamide, Topo: topotecan, *: bone marrow not assessed at this time point based on clinical judgment. **b)** Allele-specific expression of *BARD1* in tumor RNA-seq of SJNBL031647. In addition to S179_Y180fs mutation (highlighted in red), four heterozygous SNPs (labeled SNP1–4) identified from the germline exome were used for this analysis; each site had ≥5X coverage in RNA-seq. Their variant allele fraction (VAF) values in germline DNA, tumor DNA, tumor RNA-seq and purity-adjusted tumor RNA-seq are shown in parallel, indicating allele specific expression (ASE) in tumor RNA (*p*-value = 0.015 based on simulation analysis, Supplementary Methods). **c)** *BARD1* expression (left) and HRD score (right) of the index patient (indicated by the red arrow) in comparison with 58 other neuroblastoma samples profiled by ClinGen program. The dotted line represents a HRD score threshold of 42.

To assess the genotype of BARD1 S179_Y180fs, we first obtained unique read count of wildtype and mutant allele in WES and RNA-seq bam files by running indelPost^58^, which performs indel re-alignment to ensure accuracy. This resulted a VAF of 0.5, 0.4 and 0.8 in germline DNA, tumor DNA and tumor RNA, respectively, indicating absence of LOH in tumor but a possible second hit by allelic-specific expression (ASE). To further investigate ASE, we incorporated 4 germline heterozygous SNPs at *BARD1* locus (chr2: 215590370–215674407, GRCh37) with ≥5 RNA-seq reads. These SNPs (i.e., rs2070096, rs2229571, rs2070094, and rs5020511) were identified by GATK HaplotypeCaller (ver. 4.0.2.1) ^59^ analysis of WES bam files. Lack of second hit in DNA has also been verified by exome CNV analysis described below. By contrast, ASE was detected in all 4 SNPs along with the mutation site (Fig. 1B).

We used a binomial distribution model to evaluate the significance of ASE in S179_Y180fs and the four SNPs assuming a DNA VAF of 0.5, which was modelled by binomial distribution: $\sum_{SNPs}\;|binom(cov,p)/cov-0.5|$, *where cov is RNA coverage for each SNP and* p=0.5. The observed RNA VAF deviation of the five variants was similarly calculated as follows.


$$\sum_{SNPs}\;\left|RNAVAF_{observed}-0.5\right|=1.264$$

We then performed a simulation by running the binomial model 1,000 times and found only VAF deviation >1.264 occurred 15 times, which gives a significant *p*-value of 0.015 for the observed ASE. Histology-based VAF adjustment with tumor purity supported the monoallelic expression of the mutant allele tumor, which was performed as:

$$VAF_{adjusted}=\left\{VAF_{observed}-0.5\cdot (1-purity)\right\}/purity$$

### Homologous Recombination Score

We ran scarHRD^60^ to score HRD in neuroblastoma tumors. WES of our index sample was analyzed along with 58 other neuroblastomas profiled by ClinGen during 2016–2019. The somatic CNV files used as the input for scarHRD were computed from WES by running Sequenza^36^. In this cohort, our ClinGen pipeline reported two additional cases with P/LP germline mutations amongst the HRR genes involved in homologous recombination (i.e. *ATM, ATR, BAP1, BARD1, BLM, BRCA1, BRCA2, BRIP1, CHEK1, CHEK2, CDK12, FANCA, FANCC, FANCD2, FANCF, MRE11A, NBN, PALB2, RAD50, RAD51C, RAD51D,* and *WRN)* ^61^. Both mutations affect *CHEK2* and are heterozygous in tumor with a VAF of 0.46 and 0.48, respectively.

## Data Availability

Whole-exome and transcriptome sequencing datasets were obtained from St Jude Cloud which host genomic data and limited clinical information from patients/guardians who provided written informed consent^57^. The data were obtained by querying neuroblastoma under accession SJC-DS-1004 and SJC-DS-1007 which represents Genome4Kids and real-time clinical sequencing protocols, respectively. Quality control and mapping of sequencing dataset was performed through the clinical genomics pipeline as described previously^30^.
